# Divergence and hybridization in sea turtles: Inferences from genome data show evidence of ancient gene flow between species

**DOI:** 10.1111/mec.16113

**Published:** 2021-08-30

**Authors:** Sibelle Torres Vilaça, Riccardo Piccinno, Omar Rota‐Stabelli, Maëva Gabrielli, Andrea Benazzo, Michael Matschiner, Luciano S. Soares, Alan B. Bolten, Karen A. Bjorndal, Giorgio Bertorelle

**Affiliations:** ^1^ Department of Life Sciences and Biotechnology University of Ferrara Ferrara Italy; ^2^ Department of Sustainable Agro‐ecosystems and Bioresources Fondazione Edmund Mach Trento Italy; ^3^ Natural History Museum University of Oslo Oslo Norway; ^4^ Archie Carr Center for Sea Turtle Research and Department of Biology University of Florida Gainesville FL USA

**Keywords:** Chelonioidea, demographic history, gene flow, hybridization, marine turtles, whole genomes

## Abstract

Reconstructing past events of hybridization and population size changes are required to understand speciation mechanisms and current patterns of genetic diversity, and ultimately contribute to species' conservation. Sea turtles are ancient species currently facing anthropogenic threats including climate change, fisheries, and illegal hunting. Five of the seven extant sea turtle species are known to currently hybridize, especially along the Brazilian coast where some populations can have ~32%–42% of hybrids. Although frequently observed today, it is not clear what role hybridization plays in the evolutionary diversification of this group of reptiles. In this study, we generated whole genome resequencing data of the five globally distributed sea turtle species to estimate a calibrated phylogeny and the population size dynamics, and to understand the role of hybridization in shaping the genomes of these ancient species. Our results reveal discordant species divergence dates between mitochondrial and nuclear genomes, with a high frequency of conflicting trees throughout the nuclear genome suggesting that some sea turtle species frequently hybridized in the past. The reconstruction of the species' demography showed a general decline in effective population sizes with no signs of recovery, except for the leatherback sea turtle. Furthermore, we discuss the influence of reference bias in our estimates. We show long‐lasting ancestral gene flow events within Chelonioidea that continued for millions of years after initial divergence. Speciation with gene flow is a common pattern in marine species, and it raises questions whether current hybridization events should be considered as a part of these species' evolutionary history or a conservation issue.

## 1 INTRODUCTION

Speciation in the open ocean is a complex process as few natural barriers to gene flow are present compared to terrestrial habitats. Examples of marine animals that speciated as a consequence of allopatry (i.e., geographical isolation) are not as frequent when compared to terrestrial animals, as many of them have a panmictic worldwide distribution, large dispersal capacity, and less pronounced geographical barriers (Faria et al., 2021; Palumbi, 1992). Comparisons of clades of species that occupy marine and freshwater habitats showed that speciation rates were proportionally higher in freshwater habitats, possibly as a consequence of allopatry (Seehausen & Wagner, 2014; Wiens, 2015). Although marine environments have suffered large extinction events (Joyce et al., 2013), the impact of extinction on species’ biodiversity and their subsequent recovery may be more apparent in some marine clades than others (Carrete Vega & Wiens, 2012), as most speciation events in the marine environment are thought to be a balance between the formation of reproductive barriers between species and gene flow (Faria et al., 2021). The analysis of whole genome sequences is increasingly showing that speciation in marine organisms is a long‐term process, with gene flow occurring both in the past and in the present (Árnason et al., 2018; Westbury et al., 2020), and introgression being sometimes differentially distributed across the genome (Nikolic et al., 2020).

Sea turtles (superfamily Chelonioidea) are ancient species with interesting evolutionary characteristics such as natal homing (philopatry), oceanic migrations between feeding and rookery areas, long‐generation times, and low‐metabolic rates (Bowen & Karl, 2007). Seven species are currently recognized: the loggerhead *Caretta caretta*, the hawksbill *Eretmochelys imbricata*, the olive ridley *Lepidochelys olivacea*, the Kemp's ridley *L. kempii*, the green turtle *Chelonia mydas*, the flatback *Natator depressus*, and the leatherback *Dermochelys coriacea*. Among these, the loggerhead, the hawksbill, the olive ridley, the green turtle, and the leatherback have a worldwide distribution. They are mostly found in tropical and subtropical waters, with occasional reports of the leatherback from as far north as the Arctic circle (Bowen & Karl, 2007; Willgohs, 1957). The other two species, the flatback and the Kemp's ridley have a restricted distribution range, with the former occurring only in Australia and the latter almost exclusively in the Gulf of Mexico. All sea turtle species, excluding the data‐deficient flatback, are of conservation concern (vulnerable, endangered, or critically endangered), with globally or locally declining population sizes (IUCN, 2014). Populations from different ocean basins are genetically differentiated (Dutton et al., 1999; Hahn, 2011; Jensen et al., 2019; Reid et al., 2019; Vargas et al., 2016) and, at least for the loggerhead, the olive ridley and the green turtle, the Atlantic populations harbour less genetic diversity than Indo‐Pacific populations (Duchene et al., 2012; Dutton et al., 1999; Jensen et al., 2019; Reid et al., 2019). This difference suggests that the former originated from the latter and lost diversity during their geographic expansions (Baltazar‐Soares et al., 2020; Dutton et al., 1999; Hahn, 2011). Furthermore, recent demographic declines related to anthropogenic factors may have left an additional signature on genetic variation patterns (Rodríguez‐Zárate et al., 2013).

Sea turtle species have originated in the late Jurassic (Joyce et al., 2013; Naro‐Maciel et al., 2008). The initial speciation within this group separated Dermochelyidae (today represented only by the leatherback) from Cheloniidae. Fossil and molecular calibrations based on mitochondrial DNA (mtDNA) or few nuclear loci indicate that this event occurred around 100 million years ago (Duchene et al., 2012; Naro‐Maciel et al., 2008). Despite their long divergence time, all extant sea turtles have the same chromosomal number (2n = 56) and synteny between chromosomes is found not only within the Chelonioidea superfamily but it extends to the entire Testudines, including freshwater and terrestrial turtles (Lee et al., 2020). This is likely a consequence of the low mutation and evolutionary rates in turtles (Avise et al., 1992; Lee et al., 2020). In turn, the slow‐paced evolution may delay the onset of genomic incompatibilities after divergence, and this hypothesis is supported by the fact that turtle species with overlapping geographic distributions often hybridize (Buskirk et al., 2005; Fritz et al., 2008; Karl et al., 1995; Vilaça et al., 2012). In sea turtles, so far six hybrid combinations have been found between five species (olive ridleys, Kemp's ridleys, loggerheads, hawksbills and green turtles) and in diverse parts of the world including Canada, Brazil, Japan, and Australia (Brito et al., 2020). No in‐depth population studies have been performed in all six hybrid combinations to assess if they are fertile beyond first generation (F1) hybrids. Hybrids are generally rare, but exceptions exist. The largest nesting population of hawksbills in Brazil is remarkably formed by 32%–42% of hybrids between hawksbills and loggerheads and, to a smaller proportion, between loggerheads and olive ridleys (Soares et al., 2018; Vilaça et al., 2012). This event of frequent interspecific hybridization is possibly driven by the population decline that reduces the chance to find a mate of the same species combined with a temporal overlap in nesting season between the three species (Soares et al., 2017, 2021; Vilaça et al., 2012). F1 hybrids can backcross with both parental species and produce viable offspring (Soares et al., 2017, 2018), supporting the idea that the genome mixing that occurs today may have long term consequences. However, later generation hybrids among adult individuals have not been found yet (Arantes et al., 2020), and it is not known how frequently hybridization may have occurred in the sea turtles’ evolutionary past.

Here, we used whole genome resequencing data to investigate the phylogenetic history, population dynamics, genetic diversity, and ancient hybridization in the five globally distributed species of sea turtles. Our main goals were: (i) to reconstruct and calibrate the turtles’ mitochondrial and nuclear phylogenies and compare them to provide a robust framework of the evolutionary relationships between these species, (ii) estimate descriptive indices of genomic variation and the long‐term dynamics of effective population size in each species, (iii) use these analyses and additional specific approaches to identify signatures of past hybridization events.

## 2 MATERIALS AND METHODS

### 2.1 Sampling and laboratory work

This study is based on whole genome resequencing data of five sea turtle species. For three of them, olive ridley, loggerheads and hawksbills, tissue samples were obtained from individuals sampled in Brazil. The loggerhead and hawksbill were sampled at Praia do Forte, Bahia state, while the olive ridley was sampled at Sergipe state. All three samples were sequenced in previous studies at the mtDNA and one nuclear locus (Soares et al., 2018, 2021). At these markers, combined with nuclear sequences from their hatchlings, telemetry, isotopic niches, and morphological data, they did not show any signature of recent hybridization, but hybrid individuals had been observed in their area of origin (Soares et al., 2018, 2021). In addition, we downloaded from GenBank Illumina raw reads for leatherback (sampled from a feeding area off the Atlantic coast of Canada, SRR9074980) and for Pacific green turtle (SRR8616914). As an outgroup, we included sequences from the snapping turtle, *Chelydra serpentina* (SRR9043038).

We extracted genomic DNA from tissue samples using DNeasy Blood and Tissue Kit (Qiagen) and determined the DNA concentration of samples using the Qubit dsDNA quantitation assay. Libraries were prepared using the NEBNext DNA Library Prep Kit following the standard manufacturer's recommended protocol. Libraries were prepared and sequenced by Novogene and run in an Illumina NovaSeq 6000 using 150 bp paired‐end read chemistry. We obtained a total of 684,833,766 paired‐end reads for the three species.

### 2.2 Bioinformatics, SNP and genotype calling

Raw reads were trimmed for adapters using adapterremoval v2.2.2, discarding reads shorter than 20 bp and with quality lower than 30. Read quality per sample was assessed using fastqc v0.11.8 (Andrews, 2010) and multiqc v1.7 (Ewels et al., 2016). All trimmed reads were mapped using bwa mem v.0.7.15 (Li & Durbin, 2009) to an updated version of the de novo green turtle reference sequence (Wang et al., 2013) scaffolded to chromosomes through Hi‐C libraries (Dudchenko et al., 2017) (available at https://www.dnazoo.org/assemblies/Chelonia_mydas). Misaligned reads in proximity of indels were identified and realigned using gatk v3.8 (McKenna et al., 2010). Alignments were sorted, compressed and indexed using samtools v1.9 (Li et al., 2009). picard tools v2.18.20 (Broad Institute, 2009) was used to remove reads that were PCR or optical duplicates and for final bam validation.

SNP calling was performed with gatk for each scaffold using the HaplotypeCaller algorithm. We selected scaffolds with at least 500 kbp, which correspond to 29 scaffolds (1.95 Gb) covering 95% of the reference genome. One scaffold (Scaffold 240) was excluded as no reads mapped to it. Variants emitted by GATK were hard filtered excluding entries matching at least one of the following criteria: not a biallelic SNP, a SNP phred quality score (QUAL) <60, a significant Fisher strand test (FS > 60), a variant confidence/quality by depth (QD) <2, a RMS mapping quality (MQ) <40, a MQRankSum < –20 and a significant read position bias (ReadPosRankSum < −8.0). We additionally filtered out any variant in genomic regions showing low or excessive coverage (±4× the average genome coverage across all samples) or within 5 bp from called indels (reporting a QUAL >60).

The reference mitogenome from each species was used to map the mtDNA reads applying the same pipeline described above (mtDNA references: NC_028634, NC_012398, NC_016923, NC_000886, JX454992). SNP calling for the mitogenome was performed using samtools mpileup v1.9 (Li et al., 2009).

The data was further filtered based on alignments to repetitive regions and mappability. Repetitive regions in the de novo green turtle reference sequence were masked using repeatmasker v4.1 (Smit et al., 2015) with the Testudines database as input. Mappability was calculated using genmap 1.3.0 (Pockrandt et al., 2020) with a k‐mer length of 150 and maximum of four mismatches. A final set of regions passing filters were considered by merging all the aforementioned quality filters using bedtools intersect v2.19.1 (Quinlan & Hall, 2010), which yielded a total of 1,652,718,345 “callable” bases and corresponded to approximately 90% of our selected scaffolds.

SNP phasing was initially performed by whatshap 0.18 (Martin et al., 2016) considering the physical linkage between variants in raw reads, followed by statistical phasing with shapeit4 (Delaneau et al., 2019), setting the “‐‐use‐PS” and “‐sequencing” flags.

The annotation for the green turtle was obtained by lifting the annotation from the first draft version of the green turtle genome (Wang et al., 2013) to the Hi‐C reference, using the software liftoff (Shumate & Salzberg, 2021) with default parameters. The r package genomicfeatures (Lawrence et al., 2013) was used to extract intronic sequences in bed format for further diversity analysis.

### 2.3 Genomic variation

Heterozygosity was estimated per sample as a proxy for species‐level genetic diversity. Heterozygosity estimates within exons, within introns, and genome‐wide were computed using vcftools v0.1.15 (Danecek et al., 2011) on called SNPs using a minimum depth of 5 and a window size of 100 kbp. Mapping statistics were estimated using samtools flagstat and picard v2.24.1 CollectAlignmentSummaryMetrics. We assessed the completeness of our data set by running a BUSCO analysis with the Vertebrata and Sauropsida data sets (Simão et al., 2015). We also estimated the genome‐wide pairwise distance between all species with ANGSD, using the mapped bam files, a consensus approach (‐doIBS 2), and the following parameters: ‐minq 20 ‐minmapq 20 ‐uniqueonly 1 ‐docounts 1 ‐makematrix 1.

Recent studies have indicated that resequencing studies might suffer from biases derived from mapping to a divergent reference genome (Günther & Nettelblad, 2019). Although this might be exacerbated when analysing short reads and low coverage genomes (Günther & Nettelblad, 2019), estimates from high coverage genomes that depend on genome‐wide heterozygosity (e.g., Runs of Homozigosity, genetic diversity; Prasad et al., 2021) might also suffer from such biases. To test if our genome‐wide statistical analyses were subject to reference bias, we additionally mapped our sea turtle sequences to the leatherback reference genome (GCF_009764565.2). We ran the hPSMC analysis and *D*‐statistics (see below for description) using the leatherback reference genome to test if these analyses might also suffer from reference bias.

### 2.4 Phylogenies

We estimated species divergences under a phylogenetic framework using both mitochondrial and nuclear data. Two data sets with identical taxon sampling were assembled: the five globally distributed Chelonioidea sea turtles plus the snapping turtle as the outgroup. The first data set was the mitochondrial data set and included the whole mtDNA genome (total length: 16,700 bp ± stdev 56.34 bp). The nuclear dataset included a random subsampling (10%) of all exons (total length: = 17,734 exons and 3,662,655 bp). Exons were sampled proportionally to the length of the scaffolds to analyse an unbiased representation of the genome. To compare the estimates of our exon set with a set of strictly orthologous genes, we also extracted exons within BUSCO genes. We searched for BUSCO single‐copy orthologs using the Sauropsida data set and extracted exons with at least 80% overlap with a BUSCO gene. For each exon, consensus sequences were obtained using bcftools consensus (Li, 2011), individually aligned and concatenated. All uncalled SNPs were considered as missing data using the ‐M flag.

We inferred phylogenies under a maximum likelihood approach using raxml (Stamatakis, 2014) employing a GTR +gamma replacement model and 100 bootstrap replicates. Divergence times between species were estimated using beast2 (Bouckaert et al., 2014). We used the birth‐death model as a tree prior, since it is the best fitting to the evolution of marine turtles. We calibrated two nodes using lognormal distributions based on age estimates produced by Shaffer et al. (2017): the split of Americhelydia (root) with a mean of 119.5 million years ago (Ma) and a standard deviation of 0.09, and the split of the Chelonioidea with a mean of 68.4 Ma and a standard deviation of 0.15. Standard deviations were selected to approximate the 95% high posterior density (HPD) interval in (Shaffer et al., 2017). Each analysis was run for 500 million Markov chain Monte Carlo (MCMC) iterations, or until it reached convergence, sampled every 10,000 steps after a 10% initial burnin. We used tracer 1.7.1 (Rambaut et al., 2018) to visualize convergence, and it was considered to be reached when all variables had an effective sample size (ESS) >200 and a bell‐shaped posterior distribution.

For the mitochondrial data, given the high among‐sites heterogeneity of the replacement and compositional pattern in animal mitochondrial data (Bernt et al., 2013), we excluded non‐coding sequences and poorly aligned positions, and partitioned the remaining codon positions in four categories: first codon position, second codon positions, four‐fold degenerate sites, and remaining third codon positions. All partitions were unlinked, the replacement models for each of the partitions were defined using the bmodeltest package (Bouckaert & Drummond, 2017), and a lognormal relaxed clock and Birth‐Death tree prior were assumed. We used the model averaging approach for the substitution model since the beast2 analyses on these data sets using the GTR+G could not reach the convergence and results were not reproducible. To check for the effect of site sampling and replacement model on posterior estimates, we further analysed the whole mitochondrial data using both the GTR+G model and model averaging. In both cases the posterior divergences were compatible with those of the partitioned analysis. To investigate possible saturation in older nodes, we also analysed only the first codon positions, with the same parameters as described previously.

For the exon data set, we used a relaxed and a strict clock as priors. We could not use the relaxed log‐normal clock model for this data set since results obtained were not reproducible between runs. We further tested the effect of site sampling in the exon data set by repeating the analysis using a different set of exons, in which we only considered all exons longer than 2000 bp, or a subsample (10%) of exons longer than 2000 bp, and another set of 2389 exons (corresponding to 1857 genes) with no missing data that overlapped with BUSCO Sauropsida orthologous genes (Simão et al., 2015).

### 2.5 Demographic dynamics through time

The Multiple Sequentially Markovian Coalescent approach (MSMC2, https://github.com/stschiff/msmc2 ) was used to reconstruct the effective size trajectories in each species (Schiffels & Durbin, 2014). We used the phased variants corresponding to 1 individual (2 chromosomes) per species. Scaffolds shorter than 500 kbp were excluded as recommended (Schiffels & Wang, 2020). Effective population size and times were scaled using a mutation rate of 7.9 × 10^−9^ substitutions/site/generation (as estimated using the genomes of other reptiles: Green et al., 2014) and species‐specific generation times. Generation times were calculated for each sample's population of origin from estimates of age to maturity and reproductive longevity, as “age to maturity + ½ reproductive longevity” (Table S1). For green turtles we used the estimate from Fitak and Johnsen (2018), as it was specifically estimated for the population of origin of our sample. Twenty bootstrap replicates per species were used to estimate confidence intervals. To assess possible biases from statistical phasing and minimum coverage, we ran the same analysis using nonphased data and minimum depths of 5×, 10×, and 15×.

### 2.6 Hybridization

Three approaches were used to identify signatures of past hybridization among species: the *D*‐statistics (Green et al., 2010; Malinsky et al., 2021), the TWISST algorithm to perform topology weighting across the genome (Martin & Van Belleghem, 2017), and the hybrid‐PSMC approach (Cahill et al., 2016).

In our first approach, we used the *D*‐statistics to calculate conflicting patterns between ancestral (“A” alleles) and derived (“B” alleles) as a mean to distinguish introgression from incomplete lineage sorting (ILS). Excesses in the “ABBA” or “BABA” patterns produce deviation from *D* = 0, supporting introgression. We used angsd (‐doAbbababa 1 ‐doCounts 1) in three species combinations (((P1,P2)P3)O) between all four Chelonioidea species, and the leatherback was set as the outgroup (O). Significant gene‐flow was considered if |*Z*|‐score >3 and was assessed using jackknifing. Because *D*‐statistics was shown to be especially susceptible to reference bias and genotype calling (diploid vs. pseudo‐haploid SNP calling) (Günther & Nettelblad, 2019), we evaluated the consistency of *D* values using both reference genomes.

Local shifts in gene tree frequencies indicate introgression, and can be identified using the phylogenetic weighting procedure implemented in twisst (Martin & Van Belleghem, 2017). Twisst quantifies the frequencies (i.e., weightings) of alternative topological relationships among all individuals within defined SNP windows across the genome. Local phylogenies were estimated in PhyML with the GTR model using nonoverlapping windows of 50 SNPs, with a minimum of 45 SNPs in each window, and the “complete” option to calculate the exact weighting of each window by considering all possible subtrees. The leatherback was set as an outgroup to reduce the number of possible topologies and because hybridization only involves the other four more recently diverged species (Vilaça et al., 2012). We removed SNPs that were exclusively present in the leatherback to avoid the use of sites not present in the ingroup. We performed a topology weighting smoothing as a locally weighted average across 1 Mb regions (loess span = 1 Mb) and searched for regions where a discordant phylogeny had higher weight then the true phylogeny.

Finally, we investigated the occurrence of gene flow after species divergence by comparing the estimated time of species divergence (based on a nuclear calibrated phylogeny) with the estimated time of gene flow termination. The time when gene flow between two species stopped or became minor can be estimated using the hybrid PSMC (hPSMC) (Cahill et al., 2016). This method creates artificial F1 hybrid genomes from pseudo‐haploid sequences. Considering that the genomes of artificial F1 hybrids cannot coalesce more recently than the time of speciation of the two parental species, hPSMC allows the inference of time since species divergence as a measure of cessation of gene flow, and of the effective population size prior to divergence (Cahill et al., 2016). We used samtools mpileup to reconstruct fasta sequences from the bam files of each species, with a minimum base quality and mapping quality of 30 and a minimum depth of 5. We haploidized the sequences by randomly picking one base for each position using pu2fa (https://github.com/Paleogenomics/Chrom‐Compare). We further estimated the hPSMC of the species pairs using a bin interval of 10 bp to account for possible saturation and “4 + 25*2 + 4 + 6” atomic intervals. An upper limit of time to most recent common ancestor (*t*) of 15 was used for hawksbills/loggerheads, five for hawkbills/green turtles and of *t* = 10 for the other pairs. We used the plotting function from the hPSMC toolkit, considering the mean generation time for each pair of species, and the same mutation rate as reported above scaled for the generation time. From these graphs, we manually estimated the ancestral effective population size (*N*_e_) before the exponential growth to be 110,000 for hawksbills/loggerheads and loggerheads/olive ridleys, 120,000 for hawksbills/olive ridleys, 170,000 for green turtles/hawksbills and 210,000 for loggerheads/green turtles and olive ridleys/green turtles. We then performed simulations using msms (Ewing & Hermisson, 2010; Hudson, 2002) in order to estimate the cessation of gene flow between each pair of species. We simulated divergence times spanning from 10 Ma until 15 Ma for hawksbills/loggerheads, from 6 Ma until 11 Ma for hawksbills/olive ridleys, from 9 Ma until 14 Ma for loggerheads/olive ridleys, from 25 Ma until 35 Ma for green turtles/hawksbills, from 35 Ma until 46 Ma for loggerheads/green turtles and from 25 Ma until 37 Ma for olive ridleys/green turtles with 1 million‐year time intervals, and used the ancestral *N*_e_ previously inferred. To test for the possible influence of reference bias in the estimates obtained with hPSMC, we estimated the hPSMC of the species pairs using reads mapped to both references considering the same bins and atomic intervals as described above.

## 3 RESULTS

### 3.1 Patterns of genetic diversity

**FIGURE 1:**
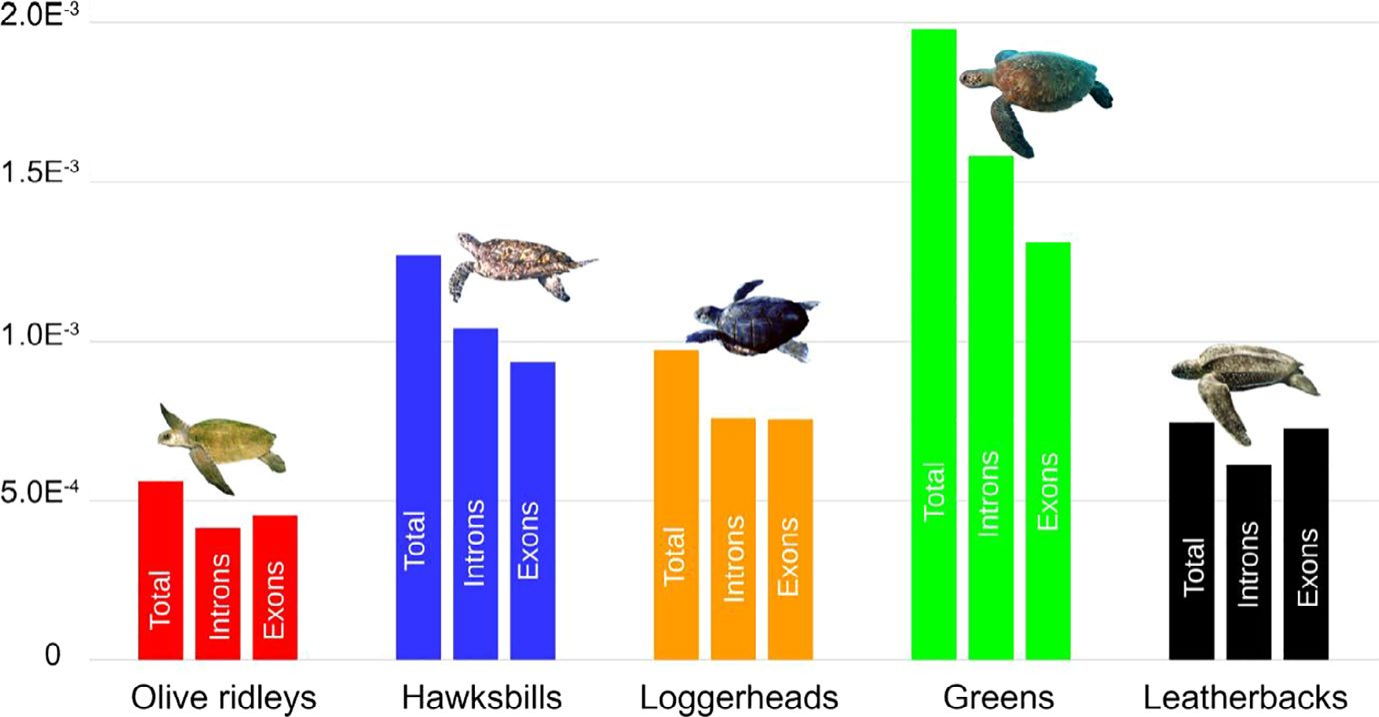
Rates of observed total heterozygosity, annotated exons, and introns per species

The number of heterozygous SNPs within species varied from 749,002 (*L*. *olivacea*) to 2,845,818 (*C. mydas*) and genome‐wide heterozygosity had a 3‐fold variation between species (Figure 1). The transition to transversion (Ts/Tv) ratio was compatible with the 2.1–2.2 value for whole genome estimates observed in other animals. All mapping statistics can be found in Tables S2 and S3. Pairwise divergence estimates showed that within Carettini species divergence is on average 1%, while this divergence is higher when considering the green turtle (~2%) and the leatherback (~4%) (Table S4).

### 3.2 Phylogenomic results

**FIGURE 2:**
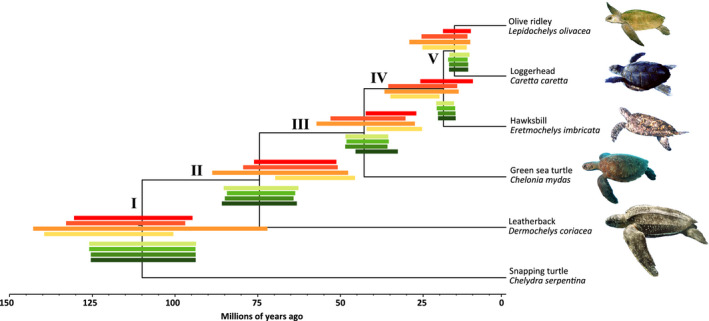
Phylogenomic relationships between sea turtle species. Bars in each node represent the divergence estimates for mitogenomes (red shades, different bars correspond to different prior distributions) and nuclear exons (green shades, different bars correspond to different prior distributions) and follow the same order as Table 1

Phylogenies based on mitogenomes for all three sets of priors estimated divergence dates congruent with previous literature. All nodes showed high support for both Bayesian and maximum likelihood trees (posterior probabilities =1, bootstrap = 100, respectively) and tree topologies were identical between the two methods (Figure 2, Figure S1). Considering all four Bayesian mitochondrial phylogenies, the divergence of leatherbacks and greens was more recent than previous estimates by an average of 40 and 20 million years, respectively, even though similar calibrations on these nodes were used for our estimates (Table 1). We also observed an overlap in the 95% HPD intervals. The divergence estimates for Carettini were similar to previous estimates and also had overlapping HPD intervals (Table 1).

**TABLE 1:** Divergence estimates for each node in the phylogenomic tree. Central values for posterior estimates are shown, followed by 95% HPD intervals within squared brackets. Estimates from two previous studies are included as comparison. Asterisks denote calibrated nodes

	Nodes	I* (Americhelydia)	II* (Chelonioidea)	III (Cheloniidae)	IV (Carettini)	V
	Prior combinations					
mtDNA	First, second, third codon sites, 4‐fold partitions	113.85 [95.9; 131.8]	64.05 [51.8; 76.3]	34.9 [27; 42.8]	20.9 [15.6; 26.2]	15.4 [11.2; 19.6]
	bModelTest partitions	115.15 [97.1; 133.2]	64.95 [50.8; 79.1]	42.65 [31.6; 53.7]	28.4 [19.9; 36.9]	20.15 [13.4; 26.9]
	GTRG4 + relaxed	108.25 [72.6; 143.9]	68.05 [48; 88.1]	43.7 [27.5; 59.9]	27.95 [16.5; 39.4]	20.2 [11.4; 29.0]
	First codon sites + strict clock GTRG4	120.35 [101.3; 139.4]	57.9 [46.1; 69.7]	34.3 [25.7; 42.9]	27.6 [20.4; 34.8]	19.5 [13.7; 25.3]
Nuclear (exons)	10% exons + strict clock	110.7 [94.0; 127.4]	76.5[65; 88]	40.5 [34.4; 46.6]	18.3 [15.6; 21.0]	15.5 [13;17.5]
	10% exons (>2000 bp) + strict clock	108.9 [92.6; 125.3]	75.7 [64.3; 87.0]	39.8 [33.8; 45.8]	17.8 [15.0; 20.6]	15.1 [12.8;17.3]
	All exons (>2000 bp) + strict clock	108.7 [92.3; 125.0]	76.6 [65.0; 88.2]	40.0 [34.0; 46.0]	17.8 [15.0; 20.5]	18.8 [12.6;17.0]
	BUSCO exons + strict clock	109.0 [92.8; 125.2]	74.9 [63.8; 86.0]	39.5 [33.6; 45.4]	17.4 [14.8; 20.0]	14.6 [12.4; 16.8]
Published	Naro‐Maciel et al. (2008) (mtDNA 12S/16S + 5 nuclear genes)	154.27 [115.14; 193.4]	108.05 [97.18; 118.91]	63.49 [35.59; 91.38]	30.52 [16.52; 44.27]	17.96 [13.53; 22.38]
	Duchene et al. (2012) (mitogenome)	125 [100; 150]	105.79 [100.00; 111.58]	58.72 [50.00; 67.44]	NA	17.75 [15.50; 20.00]

For the nuclear phylogenies, both Bayesian and maximum likelihood topologies were similar to the mitochondrial dataset and had high node support. When using a relaxed clock, we obtained a topology incompatible with the tree topology of the RaxML analysis (Figure S2), with loggerheads and hawksbills as sister species instead of loggerheads/olive ridleys as sister species (Figure 2). Due to the lack of reproducibility under relaxed clock analyses, we employed a strict clock for all nuclear analyses. Compared to mitogenomes, estimates using nuclear exons had smaller and less overlapping 95% HPD intervals (Table 1). Within Carettini the intervals are more compact than in other nodes, with almost no overlapping 95% HPD intervals, and are skewed towards more recent estimates. On average, we obtained a 2‐ to 3‐fold reduction in the width of 95% HPD intervals between the mitochondrial and nuclear exons data sets within the Cheloniidae clade, indicating that using genome data for divergence estimates allows for a greater precision in divergence estimates. Comparing central tendency values for divergence estimates between nuclear exons and mitogenomes, we obtained older although largely similar dates for leatherbacks (17% older) and green turtles (3% older), while for Carettini the central value dates were 30% (hawksbills) and 15% (loggerheads and olive ridleys) more recent than for mitogenomes. For one prior combination (Yule+LogNormal Relaxed clock), the Bayesian phylogenies were considered as nonreproducible when running more than one replicate (results not shown).

### 3.3 Demographic history

**FIGURE 3:**
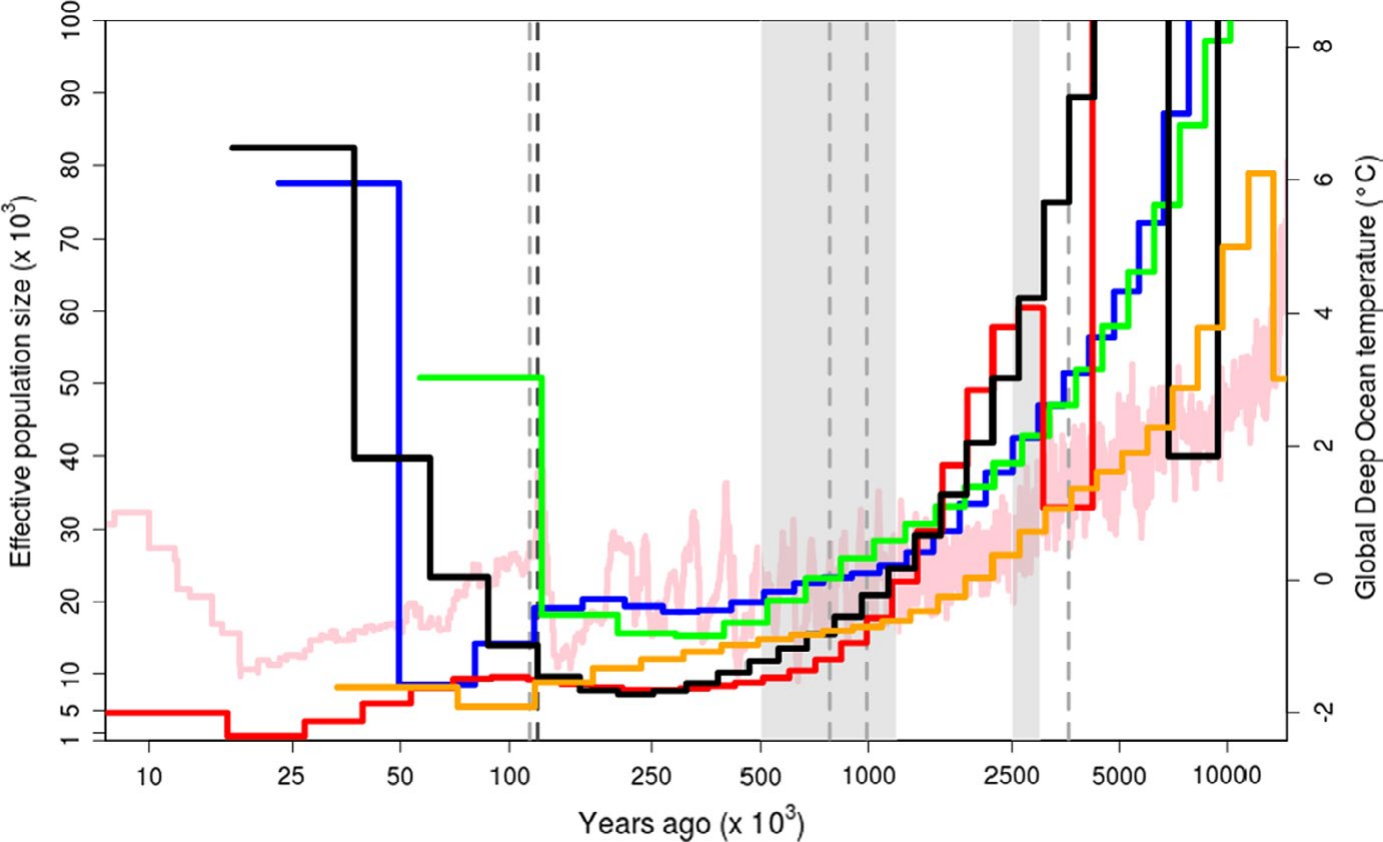
MSMC effective population size estimates with two haplotypes per species. The mid‐Pleistocene transition (MPT, 0.5–1.2 million years ago, Ma) and the Plio‐Pleistocene transition (PPT, 3.0–2.5 Ma) are shown as the light grey shaded region, major geomagnetic polarity reversals are shown as light grey dotted lines (Gilbert‐Gauss: 3.6 Ma, Brunhes–Matuyama: 0.78 Ma, Blake: 0.114 Ma; (Valet & Meynadier, 1993), and the onset of the last glaciation (0.12 Ma ago) is shown as the dark grey dotted line. The light pink line shows the global deep ocean temperatures according to (Zachos et al., 2008). Bootstrap replicates can be found in Figure S2. Red, olive ridleys; Blue, hawksbills; Orange, loggerheads; Black, leatherbacks; Green, green turtles

The reconstruction of the sea turtles’ demographies using MSMC2 spanned a total of 10 million years, covering important oceanographic events throughout time. In all species, the effective population size (*N*_e_) declined until approximately 250 thousand years ago (kya) (Figure 3). Since then, two different patterns can be identified: the loggerhead, olive ridley, hawksbill, and green turtle maintain the same trend of decline, reaching in the most recent estimate the lowest *N*_e_ throughout the sea turtles’ history; the leatherback showed a consistent pattern of demographic increase, reaching before the Last Glacial Maximum (the most recent time producing an estimate from a single individual in this species) a population size comparable to that estimated before the decline. Loggerheads, olive ridleys, and hawksbills show a more or less stable *N*_e_ estimate between 500 to 100 kya. We did not find any differences in MSMC estimates between nonphased and phased data (Figure S3), demonstrating that phasing does not insert biases when analysing a single individual. The same trend is observed when varying the minimum coverage. For the leatherback, the signal of population expansion disappears when SNPs are filtered for a higher coverage (15×), possibly a consequence of the decrease in variants analysed (39% of variants retained, in contrast to 45%–50% in other species) when a more stringent coverage filter was used.

### 3.4 Specific analyses of hybridization

#### 3.4.1 *D*‐statistics

To disentangle introgression from incomplete lineage sorting, we calculated *D*‐statistics. Only triplets that are consistent with the sea turtle phylogeny (with the leatherback used as outgroup) were considered (Table 2). In each trio, positive *D* values support hybridization between P1 and P2, while negative *D* values correspond to more frequent allele sharing between P3 and P1. The values of *D* statistics varied across the two reference genomes, especially in trios that included the green turtle and the hawksbill (Table 2), although the introgression pattern was consistent between the two references. This supports the allele sharing between green turtles and Carettini (loggerheads and olive ridleys). The values of *D* using the two references were similar *D* for the ((olive ridley, loggerhead)hawksbill) trio (*D*_Green_ = 0.03, *Z* = 33.17; *D*_Leatherback_ = 0.04, *Z* = 45.71). Another consistent trio was the ((olive ridley, loggerhead)green) (*D*_Green_ = 0.03, *Z* = 38.48; *D*_Leatherback_ = 0.04, *Z* = 56.91). Taken together, our results indicate a higher sharing of derived alleles (i.e., ABBA patterns) between green turtles and loggerheads, and between hawkbills and loggerheads independent of the reference genome.

**TABLE 2:** *D*‐statistics for sea turtles' combinations generated with ANGSD using two different reference genomes. The leatherback was used as the outgroup

((P3,P2)P1)	Green turtle reference			Leatherback reference		
	*D*‐stat	SE	*Z*	*D*‐stat	SE	*Z*
((OL,LL)HH)	0.03	0.001	33.17	0.04	0.001	45.71
((OL,HH)GG)	–0.19	0.002	–125.80	−0.07	0.001	–73.40
((OL,LL)GG)	0.03	0.001	38.48	0.04	0.001	56.91
((HH,LL)GG)	0.23	0.002	147.82	0.11	0.001	109.55

Abbreviations: HH, Hawksbills; LL, Loggerheads; OL, Olive Ridleys.

#### 3.4.2 Topology distribution across the genome

**FIGURE 4:**
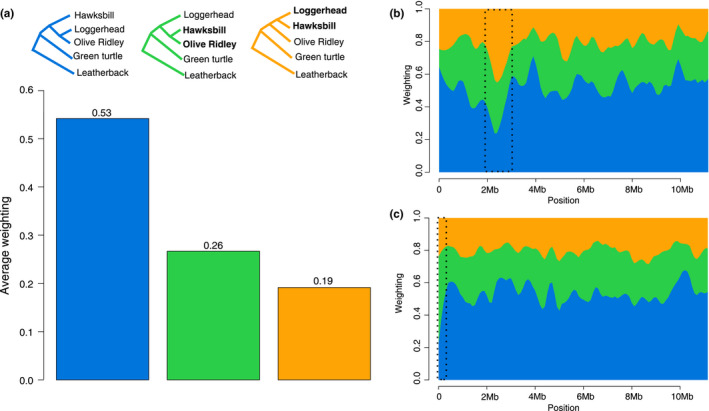
Topology weighting averages across the genomes of four sea turtle species. (a) Only the three best tree topologies are shown. Discordant species relationships are shown in bold. Distribution of topology weightings smoothed as a locally weighted average (loess span = 1 Mb) showing two regions of discordant phylogenies in the first 10 Mb of (b) scaffold 10 and (c) scaffold 3

For the 15 possible topologies of 5 species (represented as topologies rooted with the leatherback in Figure S4), TWISST showed that the most prevalent topology was identical to the species phylogeny (Figure 4, or “phylogeny topology”), followed by two phylogenetic‐discordant topologies within the Carettini tribe: one where olive ridleys/hawksbills are sister taxa and the second where loggerheads/hawksbills are sister taxa (Figure 4, Figure S4). These discordant phylogenies indicate that some genomic windows are more closely related between the two species than expected by their phylogenetic relationships, which could be a sign of hybridization between the two taxa combinations. Despite the “phylogeny topology” having the highest weighting across all topologies, it still corresponded to an overall weighting mean of 53.09 across all windows, and the other two topologies with highest weighting had an overall mean of 25.90 and 18.62, respectively (Figure 4). All other possible topologies had small weighting values (<1% each. Figure S4).

#### 3.4.3 End of gene flow

**FIGURE 5:**
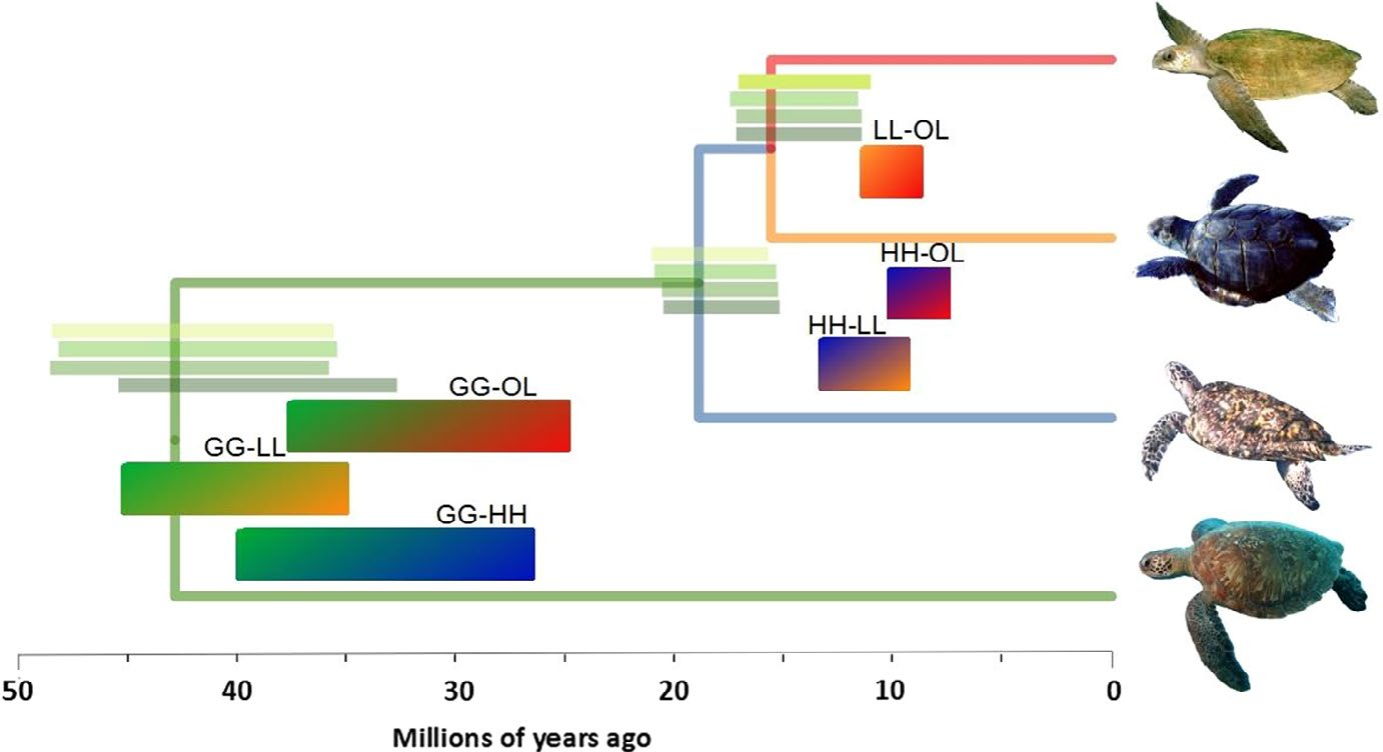
Inference of the end of gene flow between four Cheloniidae species using hPSMC (solid‐coloured bars) compared to divergence estimates based on nuclear data from the phylogeny (green shaded bars in each node). GG, green turtles; HH, hawksbills; LL, loggerheads; OL, olive ridleys

Our third analysis to look for signs of viable hybridization between Carettini species was the F1 hybrid PSMC. We did not observe any differences in the curve when mapping to the two reference genomes, and our estimates of cessation of gene flow were similar independent of the reference genome used (Figure S5). Therefore, our interpretation is based on the mapping to the green sea turtle. For the hPSMC we estimated the end of gene flow between the four Cheloniidae species. Divergence estimates were more recent for Carettini species than the mitogenomes divergences. Results from the hPSMC model and simulation analysis suggest that gene flow between hawksbills/loggerheads ceased 10–14 Ma, between hawksbills/olive ridleys 7–10 Ma, and between loggerheads/olive ridleys 9–12 Ma (Figure 5, Figure S6). When considering pairs with the green turtle, gene flow between green/loggerheads ceased 35–46 Ma, between green/olive ridleys 25–37 Ma, and between green/hawksbills 26–35 Ma These ranges, when compared with the divergence rates estimated with the nuclear genomes, point to millions of years of gene flow after divergence in Carettini species, while pairs with green turtle had an estimate cessation of gene flow that slightly overlapped with the phylogeny estimates.

## 4 DISCUSSION

### 4.1 Genetic diversity

We used whole genomes resequencing data of five sea turtle species to investigate the patterns of genetic diversity, population size dynamics and ancient hybridization between species. Relative genomic diversity across species agrees with previous estimates based on a ~1% fraction of the genome (Driller et al., 2020), with olive ridleys having one of the lowest values of genetic diversity among sea turtles and green turtles having the highest. In addition, the whole‐genome heterozygosity estimated for green turtles in a previous study (Fitak & Johnsen, 2018) is similar to the value we found. Nevertheless, we should consider that we compared greens and leatherbacks from Pacific and North Atlantic populations with loggerheads, olive ridleys, and hawksbills from the South Atlantic. The Brazilian coast is considered as an evolutionary graveyard for many marine species (Bowen et al., 2013), as it acts as a sink of biodiversity generally contributing little to overall species’ genetic richness. The Atlantic Ocean was the last ocean to be colonized by most sea turtle species, especially the Brazilian coast. Olive ridleys are the rarest species in the Atlantic Ocean and are known to have low genetic diversity, in contrast to Indo‐Pacific populations where genetic diversity of olive ridleys is much higher (Hahn, 2011), a pattern also observed in leatherbacks, hawksbills, and greens (Dutton et al., 1999; Jensen et al., 2019; Vargas et al., 2016). Therefore, future studies will probably observe higher levels of nuclear diversity in Indo‐Pacific populations (compared to the Atlantic ones), as the Indo‐Pacific seems to represent a diversification center for sea turtle species.

### 4.2 Population dynamics

Globally distributed marine species can provide important information associated with oceanic changes and worldwide processes that have affected various groups of species. In particular, studying the genomes of groups of long‐lived and slow‐evolving animals can bring important insights into ancient climate fluctuations, mass extinctions, and changes in connectivity that have had an impact in the oceans and consequently in marine animal populations. Our results showed a long term *N*_e_ decrease in three sea turtle species until 500 kya, almost perfectly matching the Pleistocene decrease in ocean temperatures (Zachos et al., 2008). The end of the sharp decrease in all species *N*_e_ coincides with the end of the Mid‐Pleistocene Transition (MPT), a period of increased ice volume, low oceanic temperatures (Clark et al., 2006), and deep ocean species extinctions (Hayward et al., 2007). After the MPT, starting approximately at 250 kya, leatherbacks showed a *N*_e_ increase, while the other species continued with the trend of decrease in effective population size reaching the lowest estimates of effective population throughout of the sea turtles’ history. The recent *N*_e_ increase of leatherbacks is consistent with a previous analysis of the French Guiana population, a likely origin of leatherbacks in the feeding areas in the Atlantic Canadian waters where our sample was collected (Molfetti et al., 2013; Stewart et al., 2013). A general pattern of expansion in greens from the Indo‐Pacific ocean was previously described using similar methods and was associated with geomagnetic polarity reversals that might have altered sea turtles’ navigational capabilities and changed gene flow patterns between populations (Fitak & Johnsen, 2018). We did not observe an increase in *N*_e_ in green turtles, as previously observed at the same period of the Brunhes–Matuyama geomagnetic reversal (circa 0.78 Ma) (Fitak & Johnsen, 2018). Even though we analysed a similar population as Fitak and Johnsen (2018), we used a slower substitution rate that was specifically estimated for reptiles (7.9 × 10^−9^ vs. 1.2 × 10^−8^ substitutions/site/generation) and a method with higher resolution in recent times than PSMC (Mather et al., 2020), which may explain the difference in the estimated demographic history. SMC‐based methods tend to lose accuracy in more recent time intervals, even if MSMC2 has been shown to better estimate recent changes than the PSMC when more haplotypes are included (Mather et al., 2020; Sellinger et al., 2020). Future studies using more individuals per population will be able to better estimate recent population size changes in sea turtles.

The population dynamics patterns exhibited by sea turtles are similar to other marine megafauna, such as baleen whales (Árnason et al., 2018), with a decrease in population size until reaching a low and relatively stable *N*_e_ between 500–250 kya. The strong decline in population sizes coincides with warmer ocean temperatures until the onset of the MPT, which caused mass extinctions and cooler global temperatures. During the MPT, many oceanic species went extinct or decreased population sizes due to changes in habitat and habitat availability (Prada et al., 2016). For sea turtles, trophic interactions like extinctions of food sources like jellyfish, algae, or sponges possibly played a role. Sponge species of the *Acropora* genus and stony corals of the *Orbicella* genus show a similar decrease in population size until the MPT (Mao et al., 2018; Prada et al., 2016), when they reached low *N*_e_ estimates around 250 kya. Sponges and corals are known to be two of the main food sources for hawksbills (León & Bjorndal, 2002) and corals form important habitats and foraging areas for sea turtle species (Becker et al., 2019). Furthermore, recent increases in global temperatures might further contribute to the decrease in population sizes, since warmer temperatures are known to decrease hatchlings’ survival (Rafferty et al., 2017; Witt et al., 2010), feminize populations (Jensen et al., 2018), and reduce coral reefs (Selig et al., 2012). It is thus likely that these species are especially vulnerable to future rapid climate warming due to a combination of historical patterns of population dynamics and current anthropogenic‐related threats.

Contrary to Cheloniidae species, leatherbacks show a gradual population expansion, possibly related to environmental changes that occurred in the last 200 thousand years. The demographic trajectories between leatherbacks and the other chelonids are very different. Leatherbacks have physiological and behavioral adaptations to cold climates, they can spend extended periods foraging in cold waters that approach 0°C, and are capable of keeping their body temperature >8°C above ambient temperature (Bostrom et al., 2010). Their physiological adaptations may have provided an advantageous expanded thermal niche allowing them to survive in a large range of ambient temperatures (James et al., 2006), favoring a demographic increase after the Pleistocene climatic oscillations in a time when many empty niches were left by extinct niche competitors. Although a *N*_e_ increase is observed in leatherbacks, they also have one of the lowest levels of genetic diversity of all sea turtles. We can hypothesize that the current genetic variation in the leatherback still reflects the Pleistocene demographic decline, and only few mutations occurred during the last 200 kya expansion. Assuming a mutation rate of 7.9 × 10^−9^ per site per generation, a generation time of 24 years, and conservatively excluding coalescent events in the single leatherback genome earlier than around 200 kya (the time of the bottleneck), less than two SNPs were produced every 10,000 base pairs since the time of the bottleneck. Therefore, factors such as long generation time and slow rate of genome evolution cause these species to take longer to restore their genetic diversity after bottleneck events (Gossmann et al., 2019).

### 4.3 Hybridization

Sea turtles are known to hybridize frequently, especially along the Brazilian coast (Brito et al., 2020; Vilaça et al., 2012). Taken together, our phylogenomic results, ABBA‐BABA patterns, topology weighting throughout the genome, and hPSMC point towards ancestral gene flow between different sea turtle species. Although previous research showed evidence of incomplete lineage sorting (ILS) in the nuclear markers of sea turtles (Vilaça et al., 2012), we show here that ancestral gene flow may have played a role in shaping their current genetic diversity.

Our nuclear phylogenies using exons showed that divergence estimates are more recent than mitogenome estimates. Although divergences in the mitochondrial tree can be younger than the species’ divergence when the mtDNA is introgressed from past hybridization, our estimates for species divergence from nuclear phylogenies were more recent than the mtDNA estimates within the Carettini. Under a pattern of divergence followed by hybridization without mtDNA introgression, estimates from nuclear genomes will be more recent than for mitogenomes, as nuclear genomes will share more variation than expected in a scenario without gene flow (Pinho & Hey, 2010). Following this rationale, our analysis using topologies across the genomes showed a high discordance in the nuclear genome, as expected when hybridization played a role in the species’ evolutionary history. Furthermore, tree priors are known to influence the divergence estimates but topologies should not be influenced by priors (Sarver et al., 2019), except when gene flow is too high and the accuracy in true topology inference decreases (Long & Kubatko, 2018). The recent divergence estimates of the nuclear genome in the Carettini tribe support the view that post‐divergence gene flow (i.e., through hybridization) might have played a role in the evolutionary history of these three species. Taking together the phylogenomic and hPSMC results, the estimates for cessation of gene flow (Figure 5) are much more recent than the divergence estimated from the phylogenies (Figure 2). Therefore, the gene flow pattern also observed in the ABBA‐BABA tests (*D*‐statistics) and hPSMC, as our results showed that gene flow continued for millions of years after divergence as estimated by the phylogenies (Figure 5), and a portion of sea turtles’ genomes are associated with hybridization events. The patterns of *D* between hawksbills/loggerheads and greens/loggerheads indicate that hybridization, albeit corresponding to a small percentage of the genome, was part of sea turtle's genome evolution. Although hybridization involving green turtles in Brazil is rarely seen (Vilaça et al., 2012), greens/loggerheads hybridize in other parts of the world (Brito et al., 2020), and ancient gene flow might have happened and left signals in the genomes of both species. Furthermore, the significant *D* values between hawksbills/loggerheads seem to be in agreement with the presence of hybrids in Brazil between these two species (Vilaça et al., 2012) and might suggest that hybridization between these two species has long been part of their evolutionary history. The addition of population data for all species will help elucidate if the same hybridization patterns are shared by different populations, and when this hybridization occurred, since gene flow might have happened before the colonization of the Brazilian coast.

If ancient gene flow indeed happened in sea turtles, we can expect that archaic introgression from extinct turtles might be present in the genomes of extant species. Turtles in general are known to frequently hybridize (Vilaça et al., 2012) and with whole genome sequences and recently developed statistical methods, the description of reticulated evolution and detection of genome fragments derived from unknown/extinct ancestors is ever more frequent (Kuhlwilm et al., 2019; Zhang et al., 2019). The contribution of an ancestral Cheloniidae or Carettini species to the genomes of hawksbills, loggerheads, and olive ridleys could also explain the discordances observed in the phylogenies, topology weighting, and *D*‐statistics. Introgression from ghost species will confound the phylogenetic signal in sections of the genome that were inherited by nonsister species, which might explain the phylogenetic discordance between the three Carettini species.

Previous investigations of current hybridization patterns from Brazil have failed to detect backcross hybrids beyond F2s between hawksbills, loggerheads, and olive ridleys (Arantes et al., 2020; Vilaça et al., 2012). Our results show hybridization within Carettini after species divergence, and since these events can still be detected in genome‐wide markers, backcrossing must have happened in the past. Even though we cannot discard the possibility that genomic incompatibilities have been developed after divergence as a species reinforcement barrier in response to hybridization, it is plausible that backcrosses survive beyond F2, even if in small numbers. The detection of gene flow between sea turtle species by our genome‐wide data indicates that signs of this ancient hybridization were inherited in the sea turtles’ genomes over many generations. Further population data for Carettini species will help elucidate divergence and hybridization patterns in sea turtles, and whether regions of the genome that show signs of hybridization had advantageous alleles and were positively selected. Our results also show that, similar to other marine species, gene flow was a part of sea turtles’ evolutionary history, and population‐level studies using whole genomes should help elucidate ancient gene flow patterns between sea turtle species.

### 4.4 Reference bias

The choice of reference genome for mapping reads from resequencing studies is a known potential source of bias, especially in estimates of heterozygosity and archaic introgression (Günther & Nettelblad, 2019), although demographic histories seem to be robust to the choice of reference (Prasad et al., 2021). Our results showed differences in estimates of *D*‐statistics, although hPSMC and *D*‐statistics patterns did not change between references. Genomes that are a mosaic of several different ancestries, which is possibly the case of sea turtles, can also affect the estimation of archaic ancestry proportions, although broad demographic conclusions when using different methods are unlikely to be biased (Günther & Nettelblad, 2019). The availability of more published data for these ancestral species can help to overcome reference bias, and possibly generate modified reference genomes to decrease mapping bias of reference/alternative alleles (Martiniano et al., 2019).

## AUTHOR CONTRIBUTIONS

Sibelle Torres Vilaça and Giorgio Bertorelle designed the research. Sibelle Torres Vilaça, Riccardo Piccinno, Omar Rota‐Stabelli, Maëva Gabrielli, Andrea Benazzo, and Giorgio Bertorelle performed the research and analysed the data. Luciano S. Soares, Karen A. Bjorndal, and Alan B. Bolten contributed samples. Sibelle Torres Vilaça and Giorgio Bertorelle wrote the manuscript. All authors contributed to writing, editing, and approved the final manuscript.

## Supporting information

Supplementary Material

## Data Availability

Raw Illumina sequences have been deposited at NCBI’s Sequence Read Archive (SRA) under BioProject PRJNA750940.
