# Supplementary material for: Divergence and hybridization in sea turtles: Inferences from genome data show evidence of ancient gene flow between species
Source: Mol Ecol. 2021 Aug 30;30(23):6178–92. doi: 10.1111/mec.16113 (PMC9292604; doi:10.1111/mec.16113)
Supplement: Supplementary file 1 — Supplementary Material [file MEC-30-6178-s001.docx]

Supporting information

**Divergence and hybridization in sea turtle species: inferences from whole genomes show evidence of ancient gene flow between species**

Sibelle T. Vilaça, Riccardo Piccinno, Omar Rota-Stabelli, Maëva Gabrielli, Luciano S. Soares, Alan B. Bolten, Karen A. Bjorndal, Giorgio Bertorelle.

Supplementary Table S1 - Generation time estimates used in this study based on population-specific data for each species.

| Species | Age to maturity (years) | Population | reference | Reproductive longevity | Population | Reference | Generation time (years) |
| --- | --- | --- | --- | --- | --- | --- | --- |
| Hawksbill | 13 (95% CI: 12−14) | Fernando de Noronha (Brazil) | Bellini et al. 2019^1^ | 14-22 | Caribbean | Mortimer & Donnelly 2008^2^ | 24 |
| Loggerhead | 31.8 (95% CI 25.7−39.2) | Brazil | Petitet et al. 2012^3^ | 28* | Espirito Santo (Brazil) | Barreto et al. 2019^4^ | 45.8 |
| Olive ridley | 14−15 | Brazil | Petitet et al. 2015^5^ | NA | NA | NA | 20** |
| Leatherback | 19 (13 - 28) | Atlantic | Avens et al. 2020^6^ | 10 (3 - 22) | Atlantic | Avens et al. 2020^6^ | 24 |
| Green turtle | 26-40 | Australia | Seminoff 2004^7^ | 19 | Australia | Seminoff 2004^7^ | 42.8^***^ |

| 1 Bellini, C., Santos, A. J., Patrício, A. R., Bortolon, L. F. W., Godley, B. J., Marcovaldi, M. A., ... & Colman, L. P. (2019). Distribution and growth rates of immature hawksbill turtles *Eretmochelys imbricata* in Fernando de Noronha, Brazil. Endangered Species Research, 40, 41-52. |
| --- |
| 2 Mortimer, J.A & Donnelly, M. (IUCN SSC Marine Turtle Specialist Group). 2008. *Eretmochelys imbricata*. The IUCN Red List of Threatened Species 2008: e.T8005A12881238. https://dx.doi.org/10.2305/IUCN.UK.2008.RLTS.T8005A12881238.en. Downloaded on 12 November 2020. |
| 3 Petitet, R., Secchi, E. R., Avens, L., & Kinas, P. G. (2012). Age and growth of loggerhead sea turtles in southern Brazil. Marine Ecology Progress Series, 456, 255-268. |
| 4 Barreto, J., Thomé, J. C., Baptistotte, C., Rieth, D., Marcovaldi, M. Â., Marcovaldi, G. G. D., ... & Cabral, L. (2019). Reproductive longevity of the loggerhead sea turtle, *Caretta caretta*, in Espírito Santo, Brazil. Marine Turtle Newsletter, 157, 10-12. |
| 5 Petitet, R., Avens, L., Castilhos, J. C., Kinas, P. G., & Bugoni, L. (2015). Age and growth of olive ridley sea turtles *Lepidochelys olivacea* in the main Brazilian nesting ground. Marine Ecology Progress Series, 541, 205-218. |
| 6 Avens, L., Goshe, L. R., Zug, G. R., Balazs, G. H., Benson, S. R., & Harris, H. (2020). Regional comparison of leatherback sea turtle maturation attributes and reproductive longevity. Marine Biology, 167(1), 4.  7 Seminoff, J.A. (Southwest Fisheries Science Center, U.S.). 2004. *Chelonia mydas.* The IUCN Red List of Threatened Species 2004: e.T4615A11037468. https://dx.doi.org/10.2305/IUCN.UK.2004.RLTS.T4615A11037468.en. Downloaded on 30 March 2021. |
| * average over three individuals reported. |
| **estimate from Abreu-Grobois, A & Plotkin, P. (IUCN SSC Marine Turtle Specialist Group). 2008. *Lepidochelys olivacea*. The IUCN Red List of Threatened Species 2008: e.T11534A3292503. https://dx.doi.org/10.2305/IUCN.UK.2008.RLTS.T11534A3292503.en. Downloaded on 12 November 2020. |
| ***estimate from Fitak, R. R., & Johnsen, S. (2018). Green sea turtle (*Chelonia mydas*) population history indicates important demographic changes near the mid-Pleistocene transition. *Marine Biology*, *165*(7), 110. |

Supplementary Table S2 - Genetic diversity estimated for sea turtle genomes mapped to the Green sea turtle genome.

|  | Olive ridley | Loggerhead | Hawksbill | Green turtle | Leatherback |
| --- | --- | --- | --- | --- | --- |
| Total reads | 263,165,997 | 233,647,915 | 358,116,164 | 848,418,489 | 481,614,874 |
| Total mapped reads (% of total) | 250,338,309 (95.13%) | 226,689,080 (97.02%) | 336,582,551 (93.99%) | 836,159,507 (98.56%) | 439,071,089 (91.17%) |
| Coverage ± Standard deviation | 19.45 ±36.27 | 17.31 ±39.31 | 21.53 ±23.60 | 41.55 ±70.99 | 21.60 ±66.62 |
| Average quality | 36.7 | 36.5 | 36.3 | 35.9 | 37.7 |
| Heterozygous sites | 749,002 | 1,337,033 | 1,795,976 | 2,845,818 | 1,100,150 |
| % Mismatches callable regions | 2.49 | 2.51 | 2.43 | 0.89 | 4.32 |
| % Mismatches introns | 2.44 | 2.46 | 2.39 | 0.83 | 4.28 |
| % Mismatches exons | 2.06 | 2.10 | 2.06 | 0.78 | 3.69 |
| Ts/Tv | 2.248 | 2.213 | 2.214 | 2.307 | 2.111 |

Supplementary Table S3 - Genetic diversity estimated for sea turtle genomes mapped to the Leatherback sea turtle genome. Numbers within brackets denote confidence intervals (CI).

|  | Olive ridley | Loggerhead | Hawksbill | Green turtle | Leatherback |
| --- | --- | --- | --- | --- | --- |
| Total mapped reads (% of total) | 92.29 | 94.09 | 89.20 | 93.21 | 96.96 |
| Coverage ± Standard deviation | 18.86 ±38.32 | 22.11 ±51.14 | 20.60 ±26.12 | 38.24 ±77.50 | 25.13 ±59.14 |
| % Mismatches | 4.41 | 4.46 | 4.40 | 4.28 | 0.006 |

Supplementary Table S4 - Pairwise distance between species calculated with ANGSD. Below diagonal: samples mapped to the Green turtle reference genome. Above digonal: samples mapped to Leatherback genome.

|  | Olive Ridley | Hawksbill | Loggerhead | Green turtle | Leatherback |
| --- | --- | --- | --- | --- | --- |
| Olive Ridley |  | 1.18% | 1.04% | 2.44% | 4.66% |
| Hawksbill | 1.16% |  | 1.09% | 2.26% | 4.26% |
| Loggerhead | 0.99% | 1.08% |  | 2.36% | 4.60% |
| Green turtle | 2.39% | 2.21% | 2.30% |  | 4.31% |
| Leatherback | 4.63% | 4.52% | 4.56% | 4.30% |  |


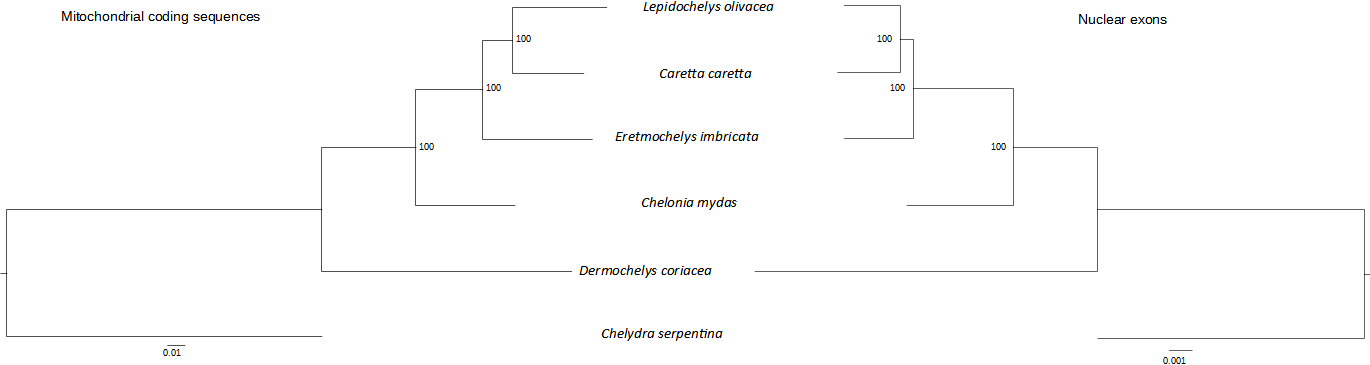


Supplementary figure S1 – Maximum Likelihood trees for mtDNA (left) and nuclear exons (right). Phylogenies were inferred under a Maximum Likelihood approach using RAxML employing a GTR + Gamma replacement model and 100 bootstrap replicates.


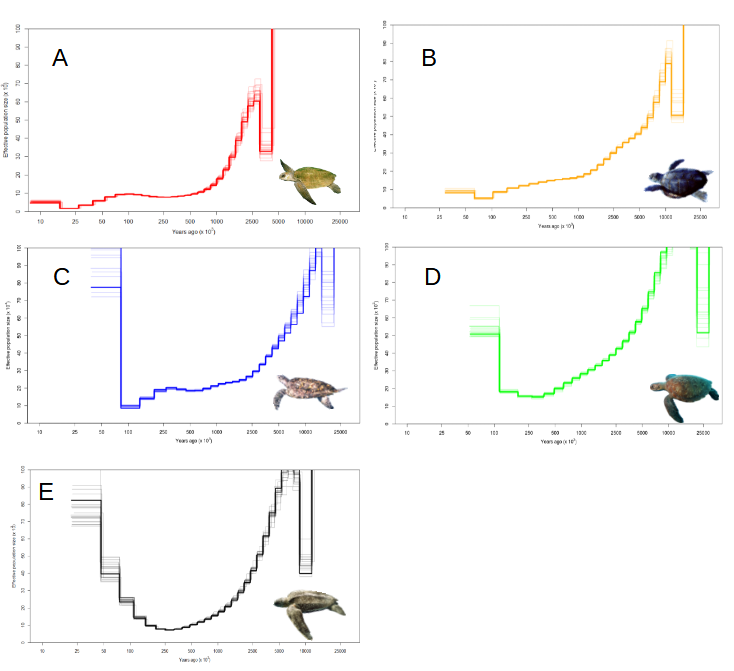
Supplementary Figure S2 - MSMC effective population size estimates with bootstrap replicates. A) Olive Ridleys; B) Loggerheads; C) Hawksbills; D) Green turtles: E) Leatherbacks.

Supplementary Figure S3 - MSMC effective population size estimates using phased and non-phased data, with varying minimum coverage.


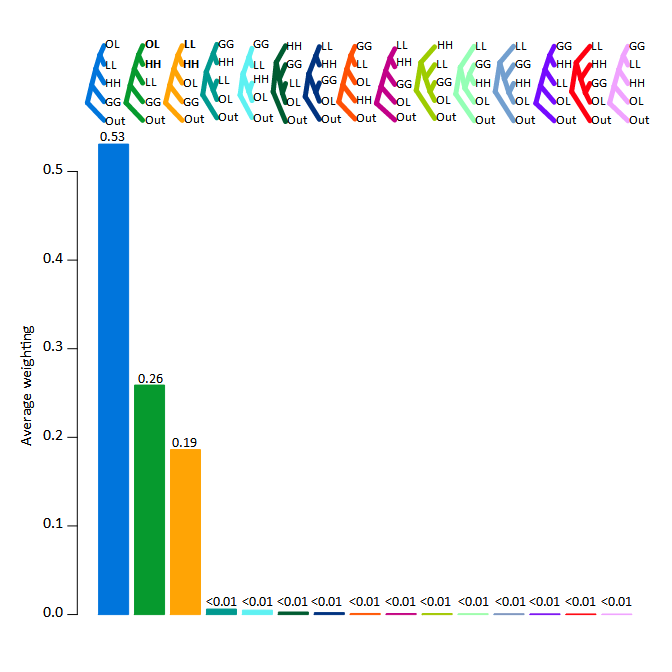


Supplementary Figure S4 - Topology weighting averages of all 15 possibile topologies across the genomes of four sea turtle species. Numbers above bars indicate average weighting across the genome for each topology. Abbreviations: Abbreviations: OL: Olive ridley, HH: Hawksbill, LL: Loggerhead, GG: Green turtle. The leatherback was used as the outgroup (Out).


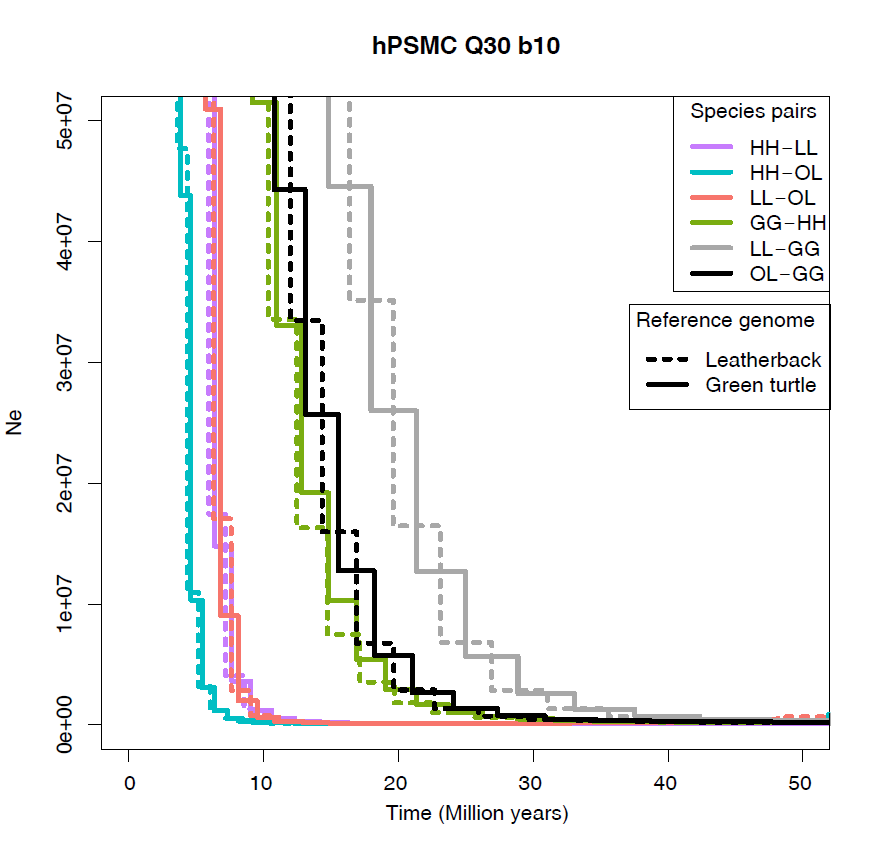


Figure S5 - Inference of the end of gene flow between four Cheloniidae species using hPSMC and two reference genomes.


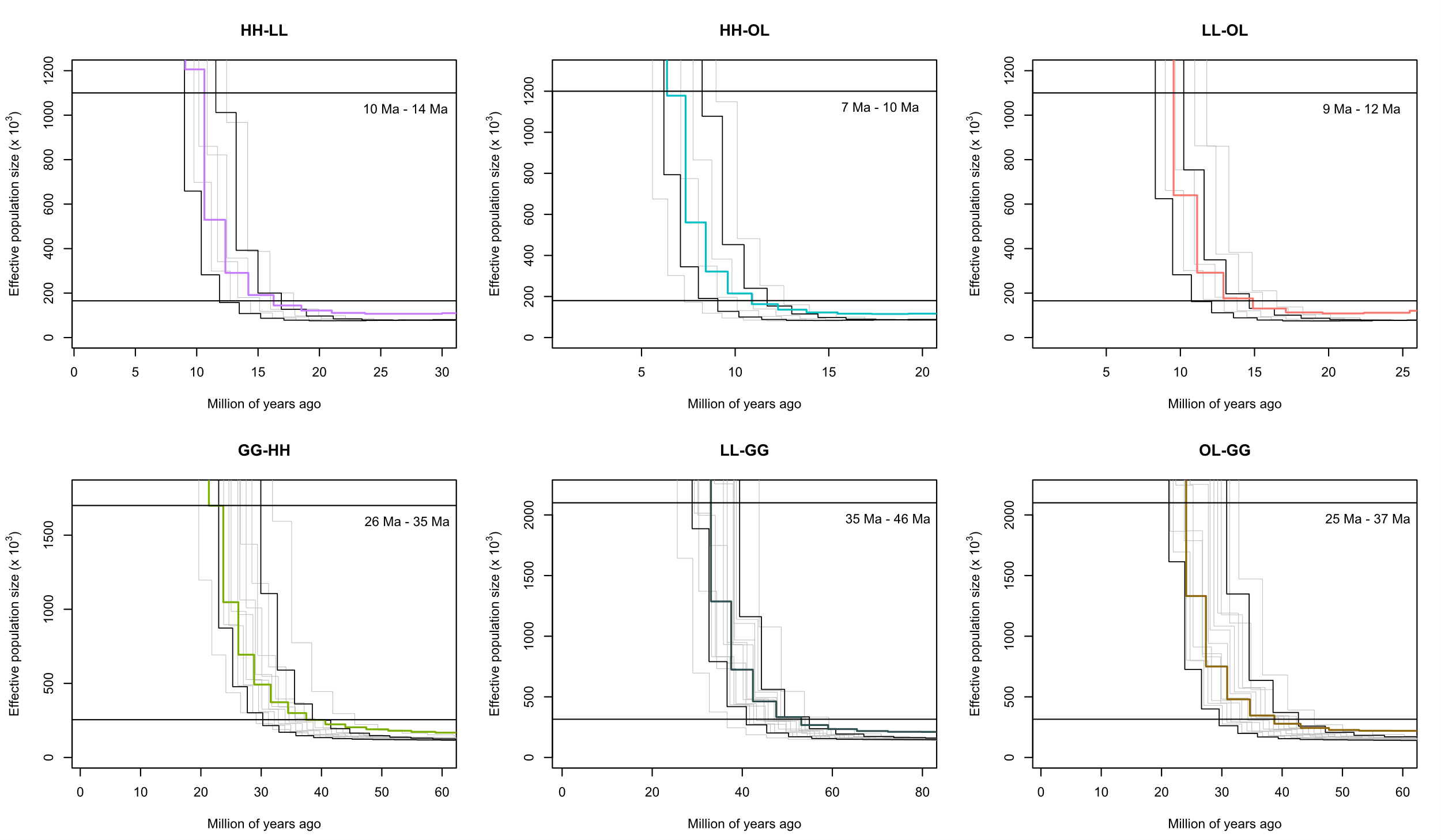
Figure S6 – Inference of the end of gene flow between four Cheloniidae species using hPSMC. Estimates for end of gene flow are shown within each graph in the top right. Colored lines represent the hPSMC results based on real data. Thin grey lines represent the simulated data, bolded black lines represent the simulations closest to the real data without overlapping it and were used to infer the time interval when gene flow ended. Abbreviations: OL: Olive Ridleys, HH: Hawksbills, LL: Loggerheads, GG=Green turtles.
